# Psychological flexibility and attitudes toward evidence-based interventions by amyotrophic lateral sclerosis patients

**DOI:** 10.7717/peerj.6527

**Published:** 2019-02-26

**Authors:** James R. Pearlman, Einar B. Thorsteinsson

**Affiliations:** Psychology, University of New England, Armidale, Australia

**Keywords:** Psychological flexibility, Relational frame theory, Amyotrophic lateral sclerosis, Motor neurone disease, Chronic illness

## Abstract

**Objective:**

Declining a percutaneous endoscopic gastrostomy (PEG) or non-invasive ventilation (NIV) by people with amyotrophic lateral sclerosis (ALS) is often contrary to advice provided by health-care-professionals guided by evidence-based principles. This study proposes relational frame theory (RFT) to offer a viable explanation of this phenomenon.

**Design:**

A total of 35 people (14 female, 21 male) aged between 34 and 73 years, with ALS, participated in this cross-sectional research.

**Main outcome measures:**

This research examined the predictive power and interaction effect of psychological flexibility (the fundamental construct of RFT) and psychological well-being on attitudes toward intervention options.

**Results:**

Participants with high psychological flexibility reported lower depression, anxiety, and stress, and higher quality of life. In addition, psychological flexibility was predictive of a participant’s understanding and acceptance of a PEG as an intervention option. Psychological flexibility was not found to be a significant predictor of understanding and acceptance of NIV.

**Conclusion:**

Although the criterion measure had not been piloted or validated outside of the current study and asks about expected rather than actual acceptance, findings suggest that applied RFT may be helpful for clients with ALS.

## Introduction

Psychological distress can affect a person’s decision-making process, leading to sub-optimal outcomes in many instances (Starcke & Brand, 2012). Amyotrophic lateral sclerosis (ALS; also referred to as motor neurone disease) is a progressive neurological disease leading to paralysis and ultimately death, most commonly as a result of respiratory failure (Bungener et al., 2005). The psychological impact on the individual can be devastating, with high levels of distress and depression being frequently reported by people with ALS (Pagnini, 2013). While there is currently no cure for the neurodegenerative process, there are intervention options available (e.g., percutaneous endoscopic gastrostomy (PEG) or non-invasive ventilation (NIV)) that have been shown to not only improve the patient’s quality of life but also potentially prolong their life (Bourke et al., 2006; Kiernan et al., 2011; Leigh et al., 2003). Despite the evidence and best-practice directives from health authorities, many ALS patients choose not to adopt these intervention options when recommended by health-care professionals (Hogden et al., 2012; Leigh et al., 2003; Orrell, 2010).

This decision-making phenomenon gives rise to further questioning regarding possible psychological factors involved. Relational frame theory (RFT) is a behaviorist approach explaining human behavior as well as internal mental processes (Barnes-Holmes et al., 2004). Research shows support for applied therapies grounded in RFT for the treatment of psychological distress in patients with long-term chronic or terminal illness, such as cancer, multiple sclerosis, and HIV (Graham et al., 2016). While there are no published randomized controlled trial results examining applied RFT and people with ALS, this paper proposes that RFT offers a viable account of understanding and acceptance of intervention options in people with ALS.

### Amyotrophic lateral sclerosis

The typical ALS patient endures pain and discomfort as a result of severe cramps and twitches; loss of mobility due to weakness and loss of muscle mass; loss of weight and possible malnutrition due to difficulties in getting food to his/her mouth, chewing and swallowing; loss of communication as speech muscles become increasingly affected; and trouble with breathing as diaphragmatic muscles weaken (Leigh et al., 2003). Malnutrition is a critical determinant in the prognosis of an ALS patient and patients who become malnourished increase their risk of death sevenfold (Kiernan et al., 2011; Limousin et al., 2010). Treatment for malnutrition in ALS patients involves the insertion of a small tube through the abdomen wall into the stomach, known as a PEG (Katzberg & Benatar, 2011). This enables caloric intake (liquid feeds and fluids) directly into the stomach, bypassing the mouth and throat (Katzberg & Benatar, 2011).

After a systematic review of non-randomized controlled trials, Orrell (2010) concluded that the use of PEGs in ALS patients has a positive effect on survival. PEGs prevent malnutrition in ALS patients and have been shown to increase mean survival time by 14-months (Limousin et al., 2010). The recommendation from peak health authorities to health-care-professionals caring for ALS patients is to offer a PEG to the patient if rapid weight loss is evident or the patient has difficulties swallowing (Katzberg & Benatar, 2011; Kiernan et al., 2011). It has been estimated that approximately 50% of patients in the US, and only 23% of patients in the UK, opt for a PEG (Bede et al., 2011). Bede et al. (2011) attributes these differences to variations between health care systems driven by complex local, financial, and cultural factors. Berlowitz et al. (2016) reported that 26% of patients opted for a PEG in their Australian-based study, and interestingly, 58% of patients who used NIV also opted for a PEG. It is presumed that the influence of health care systems also applies to the Australian data.

Non-invasive ventilation is an intervention offered to ALS patients suffering from respiratory insufficiency (Bourke et al., 2006; Kiernan et al., 2011; Leigh et al., 2003). Respiratory muscle function is a strong predictor of survival and quality of life in ALS patients (Bourke et al., 2006). As the ALS patient’s respiratory performance declines, the range of associated symptoms increases including headaches, disrupted sleep, fatigue, lack of mental alertness, and general decline in mental health (Bourke et al., 2006). NIV offers relief from these symptoms through the use of a small machine, called a bi-level positive airway pressure (BiPAP), enabling the patient to inhale larger volumes of air and exhale carbon dioxide more effectively (Eng, 2006).

Despite empirical support for NIV, the take-up remains relatively low, with reported rates of use in ALS patients in Australia at 23%, UK at 13%, and USA at 20% (Bede et al., 2011; Berlowitz et al., 2016). Bede et al. (2011) also cites differences between the US and the UK to the complex local, financial, and cultural drivers of the health care system. As with PEG prevalence rates, it would be presumed that these factors also apply to Australia.

### ALS and psychological well-being

Recent research suggests the prevalence of depressive disorders in people with ALS to be approximately 19%, and, while showing no relationship with functional impairment, depression has been shown to be strongly correlated with quality of life (Körner et al., 2015; Kurt et al., 2007; McElhiney et al., 2009; Rabkin et al., 2005).

There are no known studies investigating the relationship between general psychological well-being and decision-making in people with ALS. Depression has been shown to influence decision-making in patients with chronic illness; depressed patients being three times more likely to be non-compliant with medical advice, compared to non-depressed patients (Di Matteo, Lepper & Crogan, 2000).

### Relational frame theory and understanding and acceptance of interventions

Firstly, it should be noted that the terms *accept* and *acceptance* used throughout this paper are used in the context of RFT mental processes exclusively and not, for example, confirming the physical adoption of an intervention. A recent study by Greenaway et al. (2015) has elucidated important factors that influence ALS patients’ understanding and acceptance of interventions, including: (a) acceptance of the diagnosis and the course of the disease progression, (b) willingness to take action despite having a terminal illness, (c) acceptance of the physical need for an intervention, (d) specific fears relating to the intervention, and (e) timing-factors such as procrastination emanating from a lack of clarity about the likely course of symptoms. The present paper argues that the behaviors observed in the Greenaway et al. (2015) research can be explained by RFT, and that psychological flexibility, as the psychological construct targeted in applied RFT, should be a variable of interest. Furthermore, psychological flexibility could explain a significant portion of the variation between subjects in the understanding and acceptance of intervention options in people with ALS.

Relational frame theory is grounded in behaviorist principles and aims to move beyond the basic stimulus-response processes of classical and operant conditioning, providing a comprehensive theory of internal processes explaining human cognition, emotion, and behavior as well as offering an explanation for specific psychopathologies (Barnes-Holmes et al., 2004). The processes of RFT are technical in nature and far too extensive for the purpose of explanation in the present paper.

Psychological flexibility is the key construct of interest in RFT and is defined by two parts: (a) the processes of experiential avoidance and acceptance, requiring the individual to make contact with the present moment, and (b) the act of moving through unpleasant psychological reactions toward goals aligned with chosen values (Barnes-Holmes et al., 2004; Fletcher & Hayes, 2005; Hayes, 2016; Levin & Hayes, 2009).

Greenaway et al. (2015) and Hogden et al. (2012) highlighted *acceptance* as an influential factor for people with ALS deciding on a PEG or NIV, and the process of acceptance plays an important role in building psychological flexibility (Blackledge & Barnes-Holmes, 2009; Hayes et al., 2006; Levin & Hayes, 2009; Strosahl & Robinson, 2009). Experiential avoidance is said to present in two parts; one, the event-based unpleasant emotion; and two, the action of avoidance (Friman, Hayes & Wilson, 1998). That is, experiential avoidance manifests in actions taken to avert unpleasant emotions, thoughts, memories, or physical sensations (Levin & Hayes, 2009). RFT argues that these unwanted stimuli are a result of overly dominant comparative frames, that is, alternative mental representations or frames of reference (Levin & Hayes, 2009). RFT posits that a psychologically flexible individual has the capacity to fully accept internal events, even if unpleasant, and uses strategies (other than avoidance) to move through the discomfort.

Greenaway et al. (2015) and Hogden et al. (2012) also explained the importance of *perception of choice and control* in the context of this decision-making process. RFT claims that behavior can also be directed by strategic and values-based rules, explained by a frame of coordination between two relational networks (Barnes-Holmes et al., 2004), essentially whereby two or more mental representations are linked or explained to one’s self through a self-governed rule or value. Rule-governed behavior rests on the RFT notion of *concept-of-self*, requiring the individual not only to behave, but also to use language to assign accurate meaning to one’s own behavior (Barnes-Holmes et al., 2004). According to RFT, sound self-knowledge facilitates effective functioning but omitting the use of language to assign meaning thereby removes the process of relational framing (Barnes-Holmes et al., 2004). This would be akin to Beck’s representation of a maladaptive schema (Hulbert et al., 2011).

Psychological flexibility has been shown to have a moderate to strong relationship with general psychological well-being, including quality of life, depression, and anxiety (Hayes et al., 2006). In addition, longitudinal studies have provided support for the theory that psychological flexibility predicts mental health, and not the reverse (Hayes et al., 2006). In patients with chronic illness, Graham et al. (2016) established that applied RFT is associated with improved outcomes.

We are not aware of any published studies examining psychological flexibility in ALS patients. The only published study found of some relevance examined the longitudinal effect of mindfulness on physical functioning in people with ALS (Pagnini et al., 2015). Pagnini et al. (2015) reported that mindfulness had a positive impact on the patients’ functioning, discovering that low-mindfulness patients’ physical symptoms declined by −9.64% over a 4-month time period, while high-mindfulness patients’ physical symptoms declined by −2.88% over the same time. Mindfulness also predicted quality of life, depression, and anxiety. Relationships between quality of life, psychological well-being (depression and anxiety) and physical symptoms were reported to be non-significant.

Pagnini et al. (2015) concluded that mindfulness appeared to predict physical symptom progression in ALS patients and proposed that a mind–body interaction is the mechanism responsible. However, given that PEG and NIV interventions were not accounted for in this study, and because these interventions have been shown to influence ALS symptom progression, this proposition should be accepted with caution.

## The Current Study

The aim of the current study was to examine the relationship between psychological flexibility, psychological well-being, and understanding and acceptance of interventions in people with ALS, suggesting that applied RFT may have utility with this population. No previous study has investigated these relationships in people with ALS. The present study had two objectives: Firstly, we sought to examine the relationship between psychological flexibility and understanding and acceptance of interventions in people with ALS. Secondly, we set out to examine the role of psychological well-being within the abovementioned relationship, and investigate the possibility that psychological well-being moderates the relationship between psychological flexibility and understanding and acceptance of interventions.

*H1:* Consistent with an RFT account of feelings and behavior, we expected that high psychological flexibility would be associated with high levels of understanding and acceptance of interventions, for both PEG and NIV. In line with the literature review presented in Hayes et al. (2006), we also expected that high psychological flexibility would also be associated with high psychological well-being (i.e., high quality of life, low depression, low anxiety, and low stress).

*H2:* We expected that psychological flexibility and psychological well-being would predict understanding and acceptance of interventions.

*H3:* We expected the relationship between psychological flexibility and understanding and acceptance of interventions to be moderated by psychological well-being.

## Method

### Participants

To be eligible for this study, participants had to be aged 18-years or above and have received a diagnosis of ALS. Verification of diagnosis was not requested of the participant. The final sample consisted of 14 (40%) females and 21 (60%) males (*N* = 35), and participants’ ages ranged from 34 to 73 years (*M* = 55.80, SD = 10.48). The mean time since first symptoms was 49.14 months (SD = 32.55 months) and mean time since diagnosis was 26.74 months (SD = 21.66 months). An analysis of country of residence revealed 51.4% participants lived in Australia, 22.9% lived in the UK, 11.4% lived in the USA, 5.7% in Canada, and the remaining 8.6% in other countries. Participation was voluntary and no incentives were offered. All data for this study is available at Figshare.com (Pearlman & Thorsteinsson, 2018).

### Data screening

#### Missing data

A total of 84 participants were recruited; 31 participants withdrew from the study after viewing the information sheet; 15 participants withdrew after only completing the demographic information; and three participants withdrew after only partially completing the survey (<30% of the survey was completed). The data from these 49 participants was not included in the analysis. Missing data was addressed by calculating a mean score for each subscale (as opposed to total subscale score) and setting the denominator at a minimum of *x* − 1, where *x* was the number of items on the subscale.

### Materials

Participants were required to complete a demographics sheet which included age, gender, country of residence, date of first symptoms and date of diagnosis. Participants were then required to complete self-report measures designed to assess psychological flexibility, quality of life, depression, anxiety, stress, and understanding and acceptance of interventions.

#### Psychological flexibility

The 16-item version of the Acceptance and Action Questionnaire (AAQ-16) measures psychological flexibility across two factors; (a) willingness, and (b) action (Bond & Bunce, 2003). The first factor, “*willingness*,” includes seven items designed to measure people’s willingness to accept unwanted thoughts and feelings (e.g., “It’s ok to feel depressed or anxious”). The second factor, “*action*,” includes nine items assessing the participant’s ability to act in a way, that is, congruent with their goals and values, even in the presence of unwanted thoughts or feelings (e.g., In order for me to do something important, I have to have all my doubts worked out”). Participants responded to statements by indicating how true they believed the item was for them on a 7-point scale ranging from 1 (*never true*) to 7 (*always true*). A mean total score was calculated for each factor. Scores ranged from 1 to 7 where higher scores indicate greater psychological flexibility. Cronbach’s alpha reported by Bond & Bunce (2003) was 0.87. In the current study, the internal reliability was poor for *willingness* (Cronbach’s alpha = 0.47) and acceptable for *action* (Cronbach’s alpha = 0.69).

#### Quality of life

The McGill Quality of Life Questionnaire-Revised was designed to measure quality of life in people with a terminal illness (Cohen et al., 1995). This instrument includes 14-items to form four subscales: (1) Physical (e.g., “over the past 2 days, my physical symptoms were:”); (2) Psychological (e.g., “over the past 2 days, I was nervous or worried:”); (3) Existential (e.g., “over the past 2 days, I felt good about myself as a person.”); and (4) Social (e.g., over the past 2 days, I felt the relationships with the people I care about were:). Participants were asked to indicate an option that was most true for them on a relevantly labelled 11-point scale ranging from 0 (*very bad*) to 10 (*excellent*). Subscale scores were calculated by taking the mean score across the items in that subscale. A total mean score was calculated by determining the total for each of the 4 subscale scores. Total scores range from 0 to 10 where higher scores indicate higher quality of life. Cronbach’s alpha is 0.80 (Cohen et al., 1995). Internal reliability in the current study was good (Cronbach’s alpha = 0.88).

#### Depression, anxiety, and stress

The 21-item Depression, Anxiety, and Stress Scales was designed to assess features of depression, hyper-arousal, and tension-stress in clinical and non-clinical groups (Antony et al., 1998). Participants were presented with statements across these three subscales: (1) Depression (e.g., “I couldn’t seem to experience any positive feelings at all”); (2) Anxiety (e.g., “I was aware of dryness in my mouth”); and (3) Stress (e.g., “I found it hard to wind down”), and rate how appropriately they believed this item described them over the past week. Responses were captured on a 4-point scale ranging from 0 (*never*) to 3 (*always*). Scale scores were generated by calculating the mean total of the respective item scores, where higher scores indicate higher levels of disturbance. The Cronbach’s alphas for depression, anxiety, and stress were 0.97, 0.92, and 0.95, respectively (Antony et al., 1998). Internal reliability in the current study was good for depression and stress, with Cronbach’s alphas at 0.80 and 0.81, respectively. However, anxiety was only adequate with a Cronbach’s alpha of 0.67.

#### Understanding and acceptance of interventions

For the purpose of the present study, a 9-item scale measuring understanding and acceptance of PEGs and a 4-item scale measuring understanding and acceptance of NIV were designed. Each item was developed from qualitative findings published in Greenaway et al. (2015). Participants were required to provide self-report responses on a 5-point scale in response to questions or statements relating to their current viewpoint or attitude toward the intervention in question (e.g., PEG—“I find the idea of a PEG tube coming from my stomach…” and NIV—“I would use a BIPAP machine if it could prolong my life.”). Response options were offered on an appropriately labelled 5-point scale ranging from 1 (*highly unlikely*) to 5 (*highly likely*). Scale scores were generated by calculating the mean total (ranging from 1 to 5), where higher scores indicate higher levels of understanding and acceptance. One item on the PEG-Scale was reverse scored. The internal reliability for both scales was good (PEG-scale Cronbach’s alpha = 0.80, NIV-scale Cronbach’s alpha = 0.82).

### Procedure

Following the submission of a human research ethics application and meeting all conditions in full, the University of New England Human Research Ethics Committee approved the project (approval number HE017-069). Advertisements were placed on the Motor Neurone Disease (MND) Association website, MND Association Facebook page, and international ALS online forums. Advertisements included a hyperlink, taking prospective participants directly to the study. Data collection and storage was securely managed through online software (Qualtrics, 2017). This online capability enabled participation to people with internet access via a personal computer, tablet or mobile device. The surveys could also be completed using eye-gaze assistive-technology coupled with a tablet. The surveys took approximately 15 min to complete.

The first page presented to participants was an information sheet detailing participants’ rights and addressed ethical considerations. Participation was anonymous and participants were free to withdraw from the study at any time without consequence. Participants were then presented with a page titled “Online Implied Consent for Participant,” asking the participant to confirm that they had read and understood the information sheet and explaining that clicking on the “PROCEED TO STUDY” button served as implied consent. Withdrawal at any time could be achieved simply by closing down their web browser session. At the completion of the surveys, participants were thanked for their participation and advised that their responses had been recorded.

### Statistical analyses

Statistical analyses were performed using IBM SPSS Statistical Software version 24. A critical alpha value of 0.05 was set to test all hypotheses. Bivariate correlational analyses were conducted using Pearson product-moment coefficient calculations. A moderation analysis was performed using PROCESS (Hayes, 2018). A power analysis using G*Power 3.1 (Faul et al., 2009) was conducted based on multiple regressions in Pagnini et al. (2015). A sample size of 37 participants was recommended based on *R*^2^ = 0.15, power = 0.80, α = 0.05, Cohen’s *f* = 0.176, and six predictors.

When examining understanding and acceptance of interventions the main focus was on two factors, PEG and NIV, rather than on individual items. This was consistent with the structure of the hypothesis. However, correlations of individual items from the PEG and NIV factors and other key variables are reported in Supplementary Material (i.e., Tables S1 and S2).

## Results

### Descriptive statistics and correlations

To address the possibility of lurking variables, correlations for age, time since first symptoms, and time since diagnosis were run against all variables. Participant age was not significantly correlated with any variables measured. However, psychological flexibility (*action*) was associated with time since diagnosis (*r* = 0.45, *p* < 0.01, two-tailed) and time since first symptoms (*r* = 0.36, *p* < 0.05, two-tailed). That is, as time passes, patients are better able to take action toward goals that are in accord with their chosen values. Descriptive statistics and correlations for all predictor and criterion variables are shown in Table 1.

**Table 1:** Matrix of intercorrelations, means, standard deviations and ranges for psychological flexibility, depression, anxiety, stress, quality of life, and understanding and acceptance of interventions (percutaneous endoscopic gastrostomy and non-invasive ventilation.

Variable	1	2	3	4	5	6	7	8
1. Willingness	–	0.17	−0.27	−0.16	−0.05	0.05	0.10	0.20
2. Action		–	−0.49**	−0.45**	−0.54**	0.40*	0.59**	0.21
3. Depression			–	0.44**	0.45**	−0.60**	−0.32	−0.25
4. Anxiety				–	0.38*	−0.33	−0.32	0.19
5. Stress					–	−0.69**	−0.45**	−0.16
6. Quality of life						–	0.54**	0.27
7. UAI (PEG)							–	0.63**
8. UAI (NIV)								–
*M*	4.05	4.75	1.90	1.73	2.01	5.99	3.58	4.21
SD	0.82	0.79	0.52	0.46	0.49	1.68	0.82	0.87
Range possible	1–7	1–7	1–4	1–4	1–4	0–10	1–5	1–5
Range actual	2.43–5.86	3.56–5.86	1.00–3.14	1.00–2.86	1.14–3.14	1.17–9.02	1.89–5.00	1.00–5.00

**Notes:**

To reduce the family-wise error rate when examining these correlations without a priori hypothesis a criterion of *r* > 0.40 is suggested.

UAI (PEG), understanding and acceptance of interventions (percutaneous endoscopic gastrostomy); UAI (NIV), understanding and acceptance of interventions (non-invasive ventilation).

* *p* < 0.05, two-tailed.

** *p* < 0.01, two-tailed.

The results partly supported Hypothesis 1. As expected, results showed a strong positive correlation between psychological flexibility *(action)* and understanding and acceptance of interventions (PEG). However, no significant relationship between psychological flexibility (*action)* and understanding and acceptance of interventions (NIV) was found. Similarly, there were no significant relationships between psychological flexibility *(willingness)* and any other variable.

It was also expected that psychological flexibility would be negatively correlated with depression, anxiety and stress, and positively correlated with quality of life. This hypothesis was supported for *action*, but not for w*illingness*. Thus, these findings suggest that high psychological flexibility, specifically *action,* is associated with low depression, low anxiety and low stress, and high quality of life.

#### Multiple regression

To test the second hypothesis, a multiple regression analysis was conducted to examine the extent to which psychological flexibility and psychological well-being predicted the understanding and acceptance of interventions in people with ALS, see Table 2. The model overall was a good fit with approximately 38% of the variance in understanding and acceptance of a PEG being explained by the predictors. Table 2 shows that people with ALS who scored highly on *action* and quality of life, were expected to have a better understanding and be more accepting of a PEG as an intervention.

**Table 2:** Predicting understanding and acceptance of intervention (for percutaneous endoscopic gastrostomy) from psychological flexibility and psychological well-being.

Predictor	*b*	CI_95%_ for *b*		β	*r*	*sr^2^*
		Lower	Upper			
**Psychological flexibility**						
**Willingness**	0.04	−0.25	0.33	0.04	0.10	0.00
**Action**	0.54	0.17	0.91	0.52	0.59	0.16
**Depression**	0.35	−0.26	0.97	0.23	−0.32	0.03
**Anxiety**	−0.08	−0.65	0.50	−0.04	−0.32	0.00
**Stress**	0.16	−0.55	0.86	0.10	−0.45	0.00
**Quality of life**	0.25	0.04	0.46	0.51	0.54	0.11

**Note:**

Fit for model *R*^2^ = 0.49, Adjusted *R*^2^ = 0.38, *F*(6, 28) = 4.40, *p* < 0.01. The squared semi-partial (*sr^2^)* correlation given is the squared part-correlation given from SPSS. The *r* given is the zero-order correlation given from SPSS.

The second hypothesis suggested that psychological flexibility (*willingness* and *action*), depression, anxiety, stress, and quality of life would predict understanding and acceptance of NIV. The fit for the overall model was not significant. Table 3 shows that the relevant regression model was of a moderate strength, with 12% of the variance in the criterion being explained by the predictors suggesting that participants who were high in *anxiety* had higher understanding and acceptance of NIV.

**Table 3:** Predicting understanding and acceptance of intervention (for non-invasive ventilation)—raw data version from psychological flexibility and psychological well-being.

Predictor	*b*	CI_95%_ for *b*		β	*r*	*sr^2^*
		Lower	Upper			
**Psychological flexibility**						
**Willingness**	0.19	−0.18	0.56	0.18	0.20	0.03
**Action**	0.27	−0.20	0.74	0.25	0.22	0.04
**Depression**	−0.26	−1.04	0.52	−0.16	−0.25	0.01
**Anxiety**	0.86	0.14	1.59	0.36	0.19	0.15
**Stress**	0.11	−0.79	1.00	0.06	−0.16	0.00
**Quality of life**	0.14	−0.13	0.40	0.26	0.27	0.03

**Note:**

Fit for model *R*^2^ = 0.28, Adjusted *R*^2^ = 0.12, *F*(6, 28) = 1.80, *p* > 0.05. The squared semi-partial (*sr^2^)* correlation given is the squared part-correlation given from SPSS. The *r* given is the zero-order correlation given from SPSS. The transformed data showed the same pattern of findings (β values) but a higher Adjusted *R*^2^ (0.19), thus the more conservative raw findings are examined due to the small sample size.

#### Moderation analysis

A moderation analysis was conducted to test the third hypothesis, that psychological well-being (moderator variable) would moderate the relationship between psychological flexibility (independent variable), and understanding and acceptance of interventions (dependent variable). We therefore expected the interaction effect between the psychological flexibility and the psychological well-being to be significant upon the understanding and acceptance of interventions.

Using the consolidated data representing the three variables, the analysis was conducted using *Model 1* in PROCESS, using 5,000 bootstrapped samples (Hayes, 2018). The fit for the model overall was acceptable, *R*^2^ = 0.26, *F*(3, 31) = 5.06, *p* < 0.01. However, the interaction effect between psychological flexibility and psychological well-being on understanding and acceptance of interventions was non-significant, *b* = −0.01 [95% CI −0.60–0.57]. Thus, the results did not support the hypothesis.

## Discussion

The findings from the current study may offer some support for the position that an RFT account of behavior is tenable when evaluating the attitudes toward a PEG tube in people with ALS. Results supported the hypothesis that psychological flexibility is a significant predictor of understanding and acceptance of a PEG as an intervention for people with ALS. More specifically, the ability for an individual to take *action* in line with chosen goals and values appears most significant, more so than an individual’s *willingness* to accept unwanted thoughts or feelings. This could be helpful from a clinical perspective with results implying that applied RFT may be helpful for clients with ALS. While building acceptance and lowering experiential avoidance are generally of prime importance in applied RFT, the current study suggests that the clinician should also be cognizant of developing the client’s *action* skills, enabling the client to make better decisions throughout the course of their illness.

The data also suggested that a patient’s ability to take *action* in line with chosen goals is positively related to the time they have had ALS. This relationship may be somewhat intuitive. As time passes, patients may develop a better understanding of the nature of their disease as well as the options available to them to support their goals. Consequently, they may become better equipped to evaluate options and make decisions as time progresses. While not included in the scope of this study, the type of ALS (e.g., bulbar-onset, limb-onset) may impact the rate of symptom progression, thereby potentially exerting an influence on the point raised, and, as such, should be taken into consideration by the reader.

The results of the current research also offer some weight to the argument that the conclusions drawn in Pagnini et al. (2015)— that mindfulness predicts ALS symptoms through a mind-body interaction—may be compromised. Instead, these results offer support for the idea that mindfulness, in this case psychological flexibility, may predict a patient’s attitudes toward evidence-based treatments that prolong life. Thus, suggesting that the patient’s selection of interventions is an extraneous variable in the Pagnini (2013) research.

In line with Hayes et al. (2006), the current study found that high psychological flexibility was shown to be related to lower levels of depression, anxiety, and stress as well as higher levels of quality of life. No previous study had examined the possibility that psychological well-being moderated the relationship between psychological flexibility and understanding and acceptance of intervention options. This proposed interaction effect was shown to be non-significant and therefore offers little support for the proposed relationship. While psychological well-being did not significantly moderate this relationship, it should be noted that quality of life was a significant predictor of understanding and acceptance of a PEG in people with ALS. This relationship should be of interest to clinicians responsible for the delivery of treatment to people with ALS who may be considering intervention options.

Our parallel analysis of understanding and acceptance of NIV was less conclusive. Anxiety was the sole significant predictor, however, the direction of the relationship suggests somewhat of a paradox from an RFT perspective, with results showing a higher propensity for a participant to be more accepting of NIV when anxiety levels were high. One possible explanation for this relationship may be that the function of NIV (i.e., additional air being supplied to the patient) may be attractive to an anxious patient experiencing shortness-of-breath, hence anxiety may play a causal role. Alternatively, perhaps anxious patients are more likely to readily accept interventions that do not induce further anxiety.

Further to the analysis of understanding and acceptance of NIV, if the results observed are a true representation of this population, this would suggest that the nature of NIV decision-making may just different from PEG decision-making. Implementing NIV is: (a) far less invasive than a PEG, (b) easier to administer to the patient than a PEG, (c) a faster process and can be opted for if and when it is needed (unlike a PEG), and (d) easily reversed (unlike a PEG), should the patient wish to opt-out. On the other hand, if the results observed are not a true representation of this population, then factors including measurement error and an insufficient number of participants may also explain the phenomenon observed.

## Limitations

The criterion measure developed for this study had not been piloted or validated outside of the current study. Consequently, the implications that can be drawn from this research may be somewhat limited. Further to this point, readers should be aware that the generalizations made in the current study were based on the patient’s attitudes toward interventions, and in doing so, implying that this would be strongly correlated with the individual’s actual behavior to adopt an intervention. The validity of this assumption should be taken into consideration as a limitation.

The predicted effect size of the NIV-based criterion may have been over-estimated, and, as such, this segment of the current study had insufficient power. The reason for this smaller effect size may be down to the nature of NIV, as described previously. It should also be noted that the current study fell short of typical recommendations for the minimum number of participants, given six predictor variables, and therefore may have contributed to the study being under-powered.

The internal reliability of the measure of psychological flexibility (*willingness*) was relatively low in the current study. The relative associations may therefore have been underestimated. The literature also refers to similar shortcomings with the AAQ, and cites issues with the complexity of questions and the subtle nature of concepts addressed as potential sources of issue (Bond et al., 2011). Both of these aspects could have contributed to the internal reliability problem in the current study.

The current study recruited participants from a number of different countries. Given variation in medical practices (e.g., propensity to perform gastrostomies) and differences in patient access to resources (e.g., access to BiPAP machines though the public health system) between countries, there may be some sources of variation not accounted for in the current study design. Furthermore, the current study did not allow for any possibility that there might be some cross-cultural influence in the take-up of PEG or NIV in ALS patients. While this is an under-researched area, there is some evidence to suggest that this might be a factor (Andersen et al., 2018).

Verification of diagnosis was not sought in the current study, nor did it seek verification for type of ALS. This could potentially impact the data collected (e.g., type of ALS will be coupled with a symptom progression pattern which may, in turn, affect the patient’s decisions regarding interventions).

The scope of the current study did not include fronto temporal dementia, affecting the executive function, language and personality in 20–50% of ALS patients (Kiernan et al., 2011). Such deficits could have the potential to influence the patient’s understanding and acceptance of interventions and possibly the patient’s ability to accurately complete the questionnaire items. Finally, the cross-sectional design of this study does not allow us to make causal inferences with regard to our findings.

## Future Research

There are several directions that future research could take that are specific to this research question. The possible causal nature of the relationship between psychological flexibility and understanding and acceptance of interventions should be sought through a longitudinal research design. Alternatively, another possibility would be to investigate the role of psychological flexibility as a mediator of the relationship between education on PEGs and the understanding and acceptance of PEGs in people with ALS (Hayes, 2018; Muller, Judd & Yzerbyt, 2005). Finally, research to improve the validity of the criterion would be useful.

In a broader sense, further research into applied RFT in people with ALS is much needed. The COMMEND Project run by University College London in the UK is currently addressing this challenge in their two-phase study, customizing an RFT-based therapy for people with ALS and then assessing the treatment’s effectiveness within this group.

## Conclusion

The present study offers some support for an RFT account of behavior in explaining understanding and acceptance of intervention options in people with ALS. This research offers a first step toward developing an empirical base for applied RFT to be used with ALS patients, as it is with other chronic and terminally ill populations (Graham et al., 2016). Furthermore, the *action* factor of psychological flexibility has been highlighted to be of some importance, especially in relation to the decision to opt for a PEG. This study should serve as grounds for further research to elucidate the mechanisms involved, and to remind therapists who choose to use an applied RFT approach, of the importance of action skills as they formulate a strategy to improve psychological well-being and quality of life in people with ALS.

## Supplemental Information

10.7717/peerj.6527/supp-1 Supplemental Information 1 Matrix of Intercorrelations, Means, Standard Deviations and Ranges for Psychological Flexibility, Depression, Anxiety, Stress, Quality of Life, and Items from Understanding and Acceptance of Interventions Questionnaire (Percutaneous Endoscopic Gastrostomy.

10.7717/peerj.6527/supp-2 Supplemental Information 2 Matrix of Intercorrelations, Means, Standard Deviations and Ranges for Psychological Flexibility, Depression, Anxiety, Stress, Quality of Life, and Items from Understanding and Acceptance of Interventions Questionnaire (Non Invasive Ventilation).

10.7717/peerj.6527/supp-3 Supplemental Information 3 Intervention Understanding and Acceptance Questionnaire (NIV).

10.7717/peerj.6527/supp-4 Supplemental Information 4 Intervention Understanding and Acceptance Questionnaire (PEG).

## Additional Information and Declarations

### Data Availability

The following information was supplied regarding data availability: Pearlman JR, and Thorsteinsson EB. 2018. Adoption of evidence-based interventions by amyotrophic lateral sclerosis patients and psychological flexibility. DOI 10.6084/m9.figshare.6159182.v1.
