# Supplementary material for: Psychological flexibility and attitudes toward evidence-based interventions by amyotrophic lateral sclerosis patients
Source: PeerJ. 2019 Feb 26;7:e6527. doi: 10.7717/peerj.6527 (PMC6396741; doi:10.7717/peerj.6527)
Supplement: Supplemental Information 2 [file peerj-07-6527-s002.docx]

Table S2

Matrix of Intercorrelations, Means, Standard Deviations and Ranges for Psychological Flexibility, Depression, Anxiety, Stress, Quality of Life, and Items from Understanding and Acceptance of Interventions Questionnaire (Non Invasive Ventilation).

| Variable | 2 | 3 | 4 | 5 | 6 | 7 | 8 | 9 | 10 | 11 |
| --- | --- | --- | --- | --- | --- | --- | --- | --- | --- | --- |
| 1. Willingness | .17 | -.27 | -.16 | -.05 | .05 | .26 | .08 | .02 | .27 | .20 |
| 2. Action | - | -.49** | -.45** | -.54** | .40* | .21 | .16 | .11 | .22 | .22 |
| 3. Depression |  | - | .44** | .50** | -.60** | -.20* | -.29 | -.23 | -.13 | -.25 |
| 4. Anxiety |  |  | - | .38* | -.33 | .19 | .07 | .39* | .04 | .19 |
| 5. Stress |  |  |  | - | -.69* | -.08 | .01 | -.14 | -.26 | -.16 |
| 6. Quality of Life |  |  |  |  | - | .10 | .14 | .24 | .36* | .27 |
| 7. UAI (NIV) Q1 |  |  |  |  |  | - | .79** | .56** | .57** | .87** |
| 8. UAI (NIV) Q2 |  |  |  |  |  |  | - | .52** | .50** | .84** |
| 9. UAI (NIV) Q3 |  |  |  |  |  |  |  | - | .44** | .76** |
| 10. UAI (NIV) Q4 |  |  |  |  |  |  |  |  | - | .80** |
| 11. UAI (NIV) Total |  |  |  |  |  |  |  |  |  | - |

*Note.* UAI (NIV) = Understanding and Acceptance of Interventions (Non Invasive Ventilation). To reduce the family-wise error rate when examining these correlations without a priori hypothesis a criterion of *r* > .40 is suggested.

* *p* < .05, two-tailed. ** *p* < .01, two-tailed.
