# Supplementary material for: Psychological flexibility and attitudes toward evidence-based interventions by amyotrophic lateral sclerosis patients
Source: PeerJ. 2019 Feb 26;7:e6527. doi: 10.7717/peerj.6527 (PMC6396741; doi:10.7717/peerj.6527)
Supplement: Supplemental Information 3 [file peerj-07-6527-s003.pdf]

Intervention Understanding and Acceptance Questionnaire (NIV)

Below is a set of questions / statements relating to attitudes towards non-invasive ventilation (NIV or use of a BiPAP machine) in ALS / MND patients. Please read each question / statement below and provide a response indicating the option that you think best describes your current viewpoint (regardless of whether or not you already use one). There are no right or wrong answers. Do not spend too much time on any question.

**Question Number**	**Factor from Greenaway et al. (2015)**	**Question / Statement**			**Response**			Scoring Notes
1	Patient-Centric	If my respiratory system was heavily affected by my ALS / MND, I believe that NIV (using a BiPAP machine) could help make me feel better.	strongly disagree (1)	slightly disagree (2)	not sure (3)	slightly agree (4)	strongly agree (5)	
2	Patient-Centric	I would use a BiPAP machine if it could prolong my life.	highly unlikely (1)	unlikely (2)	not sure (3)	likely (4)	highly likely (5)	
3	Patient-Centric	How likely do you believe it will be that you will need to consider using a BiPAP machine?	highly unlikely (1)	unlikely (2)	not sure (3)	likely (4)	highly likely (5)	
4	Patient-Centric	I find the idea of using a machine to help me breathe while I sleep...	extremely disturbing (1)	very disturbing (2)	moderately disturbing (3)	slightly disturbing (4)	not disturbing at all (5)	

Scores for each item should be totalled using the scale shown. Total scores range from 0 to 20. A high total score indicates high intervention understanding and acceptance. A low total score indicates a low intervention understanding and acceptance.
