# Supplementary material for: Psychological flexibility and attitudes toward evidence-based interventions by amyotrophic lateral sclerosis patients
Source: PeerJ. 2019 Feb 26;7:e6527. doi: 10.7717/peerj.6527 (PMC6396741; doi:10.7717/peerj.6527)
Supplement: Supplemental Information 4 [file peerj-07-6527-s004.pdf]

Intervention Understanding and Acceptance Questionnaire (PEG)

Below is a set of questions / statements relating to attitudes towards gastrostomies (PEG tubes) in ALS / MND patients. Please read each question / statement below and provide a response indicating the option that you think best describes your current viewpoint (regardless of whether or not you have already opted for one). There are no right or wrong answers. Do not spend too much time on any question.

Question Number	Factor from Greenaway et al. (2015)	Question / Statement	Response					Scoring Notes
1	Patient-Centric	If I was experiencing extreme hunger and malnutrition as a result of my ALS / MND, I believe that a PEG tube could help alleviate this.	strongly disagree (1)	slightly disagree (2)	not sure (3)	slightly agree (4)	strongly agree (5)	
2	Patient-Centric	I would opt for a PEG if it could prolong my life.	highly unlikely (1)	unlikely (2)	not sure (3)	likely (4)	highly likely (5)	
3	Patient-Centric	How likely do you believe it will be that you will need to consider a PEG tube?	highly unlikely (1)	unlikely (2)	not sure (3)	likely (4)	highly likely (5)	
4	Patient-Centric	I find the idea of a PEG tube coming from my stomach…..	extremely disturbing (1)	very disturbing (2)	moderately disturbing (3)	slightly disturbing (4)	not disturbing at all (5)	
5	Patient-Centric	The thought of using a PEG tube day-to-day for my calorie intake makes feel…	extremely uncomfortable (1)	very uncomfortable (2)	moderately uncomfortable (3)	slightly uncomfortable (4)	no discomfort at all (5)	
6	Patient-Centric	Thinking about the PEG tube insertion procedure makes me…..	extremely worried (1)	very worried (2)	moderately worried (3)	slightly worried (4)	not worried at all (5)	
7	Information and Aspects of Time	I believe that it is beneficial to evaluate my options before symptoms fully present.	strongly disagree (1)	slightly disagree (2)	not sure (3)	slightly agree (4)	strongly agree (5)	
8	Information and Aspects of Time	I like to be fully informed about the progression possibilities of ALS / MND.	strongly disagree (1)	slightly disagree (2)	not sure (3)	slightly agree (4)	strongly agree (5)	
9	Information and Aspects of Time	It is hard for me to make a decision on getting a PEG tube when it's not clear whether I will need one or not.	strongly disagree (1)	slightly disagree (2)	not sure (3)	slightly agree (4)	strongly agree (5)	Reverse

Scores for each item should be totalled using the scale shown. Item 9 is scored reverse. Total scores range from 0 to 45. A high total score indicates high intervention understanding and acceptance. A low total score indicates a low intervention understanding and acceptance.
